# Antioxidant, Anti-Inflammatory and Anti-Obesity Potential of Extracts Containing Phenols, Chlorophyll and Carotenoids from Mexican Wild Populations of *Bacopa monnieri* (L.) Wettst

**DOI:** 10.3390/biology12040620

**Published:** 2023-04-19

**Authors:** Martha Martínez-García, Gloria Garduño-Solórzano, Graciliana Lopes, Begoña Astrain Sanchez, Ralph Urbatzka, Guilherme Scotta Hentschke, Jorge E. Campos, Vitor Manuel Oliveira Vasconcelos

**Affiliations:** 1 Unidad de Biotecnología y Prototipos (UBIPRO), Facultad de Estudios Superiores Iztacala, Universidad Nacional Autónoma de México (UNAM), Avenida de los Barrios No. 1, Col. Los Reyes Iztacala, Tlalnepantla 54090, Mexico; 2 IZTA Herbarium, Facultad de Estudios Superiores Iztacala, Universidad Nacional Autónoma de México (UNAM), Avenida de los Barrios No. 1, Col. Los Reyes Iztacala, Tlalnepantla 54090, Mexico; 3 Interdisciplinary Centre of Marine and Environmental Research, (CIIMAR/CIMAR), Novo Edifício do Terminal de Cruzeiros do Porto de Leixões, Avenida General Norton de Matos, S/N, 4450-208 Matosinhos, Portugal; 4 Department of Biology, Faculty of Sciences, University of Porto (FCUP), Rua do Campo Alegre, 4169-007 Porto, Portugal

**Keywords:** anti-inflammatory, anti-obesity, antioxidants, *Bacopa monnieri*, Brahmi, carotenoids, pigment profile

## Abstract

**Simple Summary:**

Using molecular markers, we confirmed the taxonomic status of *Bacopa monnieri* from Mexico. We obtained the pigment and carotenoid profiles, total phenols, antioxidants, and bioassayed the biological activities (anti-inflammatory and anti-obesity) of the four wild populations. Our results show the great potential of *B. monnieri* as a natural source of compounds with antioxidant, anti-inflammatory and anti-obesity properties. Therefore, the raw material of this plant may be effectively used as a nutritional and medicinal supplement.

**Abstract:**

Some of the species of the genus *Bacopa* have been used in Pharmacopoeia worldwide. However, in Mexico, *Bacopa monnieri* has neither been extensively cultivated nor studied, nor has their use in traditional medicine been reported. The aim of this work was to assess the taxonomic verification of the four wild populations of *B. monnieri*, the chemical content of their pigments and phenols and to provide an analysis of their potential bioactivity. *B. monnieri* wild populations from Mexico were validated using molecular markers. Chromatographic profiling using HPLC-PDA revealed 21 compounds comprising 12 chlorophylls and nine carotenoids; of the latter, the major ones were lutein (0.921 ± 0.031 μg/mg of dry extract) and β-carotene (0.095 ± 0.003 μg/mg of dry extract). The total phenolic content, determined using the Folin–Ciocalteu assay, ranged from 54.8 ± 5.8 to 70.3 ± 2.2 µg of gallic acid equivalents (GAE)/mg. Plant extracts scavenged from the free radical DPPH in IC_50_ ranged from 130.6 ± 3.0 to 249.9 ± 12.1 µg dry extract/mL. In terms of the anti-inflammatory potential, the most effective extract was from a soil-based plant from Jalisco (BS), reduced from nitric oxide in a RAW 264.7 culture medium, with an IC_50_ value of 134 µg of dry extract/mL. The BS extract showed a significant neutral lipid-reducing activity in the zebrafish model, ranging from 3.13 μg/mL *p* < 0.05 to 100 μg/mL *p* < 0.0001. Overall, the extracts analyzed here for the first time seem promising for future use because of their antioxidant, anti-inflammatory and anti-obesity potential.

## 1. Introduction

The genus *Bacopa* (Plantaginaceae) contains 107 species recorded to date [1]. Of them, *B. monnieri* (L.) Wettst. is only included in the Pharmacopoeia of the United Kingdom, the United States and India, where it is described as a medicinal resource and whose common name is Brahmi. It is the main component of various formulations on the market for medicinal use or as a food supplement [2,3]; it has a market price of approximately 3–5 USD/kg [4]. The Ayurveda system indicates its use for treating mental illness, epilepsy [5], asthma, depression, inflammatory states, oxidative stress, cancer, anxiety and as a hepatoprotective [6]. These effects are attributed to the presence of phytochemical compounds, including cucurbitacins, flavonoids, phenols, saponins and alkaloids such as brahmin and herpestine, as well as glycosides called bacosides A and B (triterpenoids similar to dammarane) and betulinic acid [3,7]. With its low toxicity risk [8] and apparently beneficial effects, *Bacopa* has been extracted and marketed as a dietary supplement [9].

The Barcode of Life [10] proposed validating biological resources, including those cited in the Pharmacopoeias, using the chloroplast genetic regions *rbc*L, *mat*K, *trn*L [11] and nuclear ITS [12]. Using a marker sequence analysis makes it possible to avoid species confusion and, therefore, to taxonomically validate plants with commercial value.

*Bacopa* biodiversity in Mexico records seven species: *B. lacertosa* Pennell *ex* Standl., *B. repens* (Sw.) Wettst., *B. rotundifolia* (Michx.) Wettst., *B. salzmannii* (Benth.) Wettst. *ex* Edwall., *B. sessiliflora* (Benth.) Edwall., *B. valerioi* Standl. & I.T. Williams and *B. monnieri* (L.) Wettst, the last being more widely distributed [13]. None of these species are cultivated in Mexico. *B. monnieri* is an herbaceous plant which grows from 0 to 3500 m asl and has a global distribution [1].

It is essential to evaluate the germplasm of wild and cultivated populations from different locations to search for elite genotypes [14], as studies in India have shown that the concentration of bacosides, commercially valued compounds, is lower in cultivated plants [15].

To improve health problems, it is necessary to search for local phytogenetic resources that have beneficial properties for the treatment or prevention of obesity, as a basis for new functional food supplements. Overweight and obesity are a growing public health problem throughout the world, including Mexico. The causes of obesity are multifactorial; however, a crucial factor is the food transition that has occurred since the late 1980s, with an increase in the consumption of processed foods and high calorie diets with high sugar and fat contents, coupled with a sedentary lifestyle [16].

*B*. *monnieri* could be a promising option for improving these health problems because of its anti-inflammatory activity; however, despite being used in other regions of the planet, this resource has not yet been included in the Mexican Pharmacopoeia. To our knowledge, no research has been conducted to date on the pigment or carotenoids profiles of the wild and cultivated populations of *Bacopa* spp. from Mexico, nor on its biological activity which may have anti-obesity and anti-inflammatory benefits, which would justify proposing *B. monnieri* as a functional food. Consequently, the objective of this work was: (i) the taxonomic verification of the four wild populations of *B. monnieri* from the Central Mexican Volcanic Belt (CMVB) based on molecular markers, (ii) the determination of the chemical content of the different *B. monnieri* extracts in terms of their pigments and phenols and (iii) the analysis of their potential bioactivity for their application to human health issues such as inflammation, obesity and oxidative stress.

## 2. Materials and Methods

### 2.1. Materials

**Figure 1:**
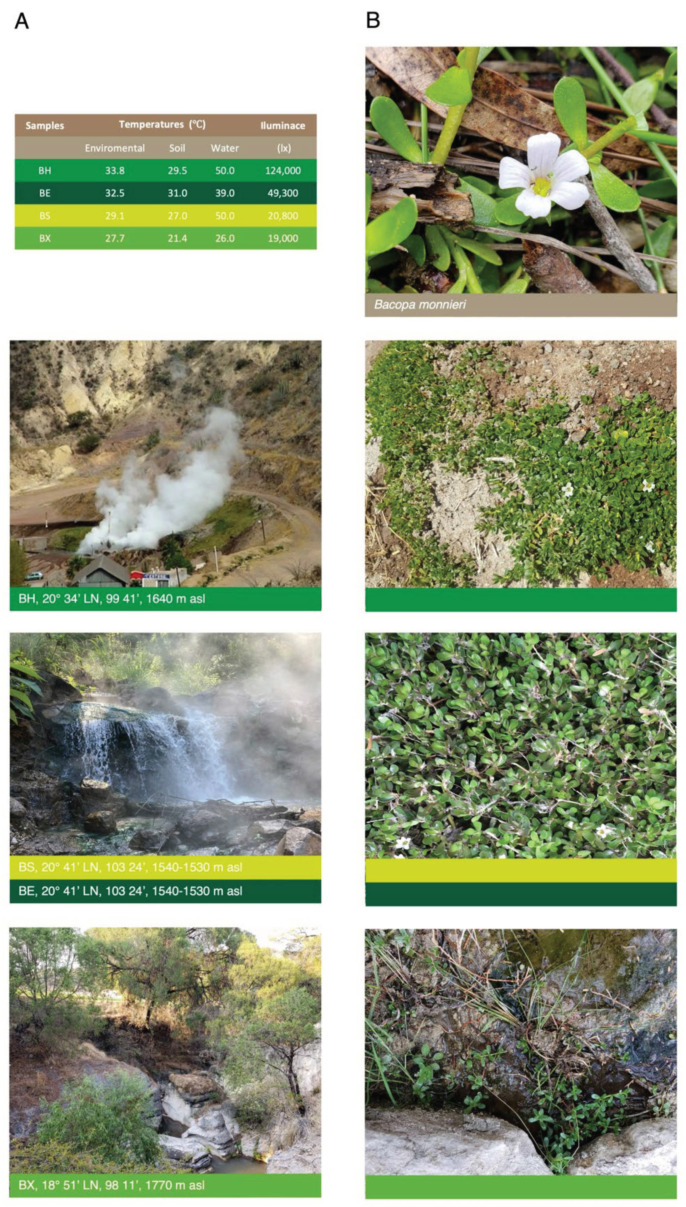
Images and collection data for the wild Mexican plant *B. monnieri.* (**A**) Environmental data and panoramic views of the three localities. (**B**) Flower details, followed by the plants in the soil at BH, BS and BX, respectively.

Wild *Bacopa monnieri* was sampled from three localities within the CMVB in 2019. The samples were geopositioned and environmental data were recorded as BH (Geiser, Hidalgo), BS (Bosque de la Primavera, Jalisco) and BX (San Felipe Xonacayucan, Puebla). They were terrestrial life forms, except for BE (Bosque de la Primavera, Jalisco), which was an emergent aquatic life form, as seen in (Figure 1). All materials were submitted to the IZTA Herbarium [17].

### 2.2. Molecular Identification

Whole plants were transported to the laboratory, frozen in liquid nitrogen and maintained at −70 °C until use. According to the manufacturer’s instructions, the complete genomic DNA of *B. monnieri* was extracted using the UltraClean Plant DNA Isolation Kit (MoBio Laboratory Inc., Carlsbad, CA, USA). For amplification of the three DNA regions defined as the universal DNA barcoding for plants, the partial gene *rbc*L, ITS and intergeneric region *trn*F-*trn*L, previously published oligonucleotides were used (Table 1).

Amplification reactions using Phire Green Hot Start II PCR Master Mix (Thermo Scientific, Carlsbad, CA, USA) were performed as follows: Initial denaturation at 94 °C for 1 min, followed by 35 cycles at 94 °C for 1 min, 50 °C for 45 s, 72 °C for 1 min, and a final 10 min extension at 72 °C, except for *trn*F-*trn*L, which had an alignment temperature of 55 °C. The PCR products were separated in a 1.5% agarose gel in a 1X TAE buffer. The samples were gel extracted and purified (NZYTech Gelpure Kit, Nzytech, Portugal). Sanger sequencing was conducted at GATC Biotech (Konstanz, Germany). The data were analyzed and edited using Geneious 9.1 (Biomatters Inc., Boston, MA, USA). GenBank accession numbers were obtained and with the data sequence a BLASTn was performed.

### 2.3. Preparation of Extracts

To explore the influence of environmental conditions on the pigment profile of *B. monnieri*, the whole plant was washed with distilled water, frozen at −70 °C and lyophilized. Samples were ground to a fine powder using a Precellys 24 homogenizer (Bertin Technologies Montigny-le-Bretonneux, France) and kept away from light until extraction.

Pigments were extracted with methanol (reagent grade). First, 2 g of the powdered sample was suspended in 50 mL of methanol and sonicated (Vibra Cell^TM^ ultrasonic liquid processor, Sonic & Materials, Inc., Newtown, CT, USA) at a frequency of 70/800 Hz for 3 min. The procedure was repeated three times and then the supernatants were combined and evaporated (165 mbar, 30 °C) (Centrivap Vacuum Concentrator, Labconco, Kansas City, MO, USA), then kept at −20 °C until pigment analysis. Before HPLC-PDA analysis, the dry extracts were resuspended in methanol (HPLC grade) to a final concentration of 10 mg/mL and filtered through a GH Polypro (GHP) Membrane Disk Filter (0.2 μm diameter) (Pall, NY, USA) [21].

### 2.4. Determination of Carotenoids and Chlorophylls Profile using HPLC-PDA

Pigment profiling for *B. monnieri* followed the methodology previously reported [21]. The extracts were analyzed in an HPLC system coupled with a photodiode array (PDA) detector (Waters Alliance 2695 Separations Module, Waters Corporation, Milford, CT, USA). The chromatographic conditions were performed according to Lopes et al. [21].

All the HPLC-grade solvents were filtered with GH Polypro (GHP) Membrane Disk Filters (0.2 μm diameter) (Pall, NY, USA). Data were analyzed using Empower chromatography software (Waters^TM^, Milford, MD, USA). Chromatograms are shown in Figure S1. Spectra data (250–750 nm) from all compounds were recorded.

The compound’s retention times and UV-Vis spectra were compared to authentic standards (Extrasynthese, Genay, France and Sigma-Aldrich, St. Louis, MO, USA). The carotenoid’s quantification was achieved by measuring the absorbance recorded in the chromatograms relative to external standards at 450 nm. Lutein and chlorophylls a, b and β-carotene were quantified with the authentic standards. The unidentified carotenoids were quantified as lutein, the chlorophyll derivatives as chlorophyll a, the violaxanthin derivatives as violaxanthin and the Ɛ-carotene derivatives as Ɛ-carotene. Calibration curves were obtained with standard solutions corresponding to five different concentrations, selected as representative of the samples’ ranges of compounds concentrations. The calibration plots, *r^2^* values, the limit of detection (*LOD =* 3*So/b*) and the limit of quantification (*LOQ =* 10*So/b*) (where *So* is the standard deviation of the signal-to-noise ratio and *b* is the slope of the calibration plot) for the analyzed carotenoids and chlorophyll a are shown in Table 2. Pigment quantity in each sample was calculated as μg/g of lyophilized biomass.

### 2.5. Total Phenolic Content

The total phenolic content (TPC) of the extracts of *B. monnieri* was determined as previously described, using the Folin–Ciocalteu assay [22]. Briefly, a volume of 25 µL of each wild *Bacopa* extract was carefully mixed with 500 µL of deionized water, 100 µL of Na_2_CO_3_ solution (75 g/L), 25 µL of Folin–Ciocalteu reagent (Sigma-Aldrich, St. Louis, MO, USA) or 25 µL of H_2_O for blanks. The reaction was conducted for 60 min. at room temperature in the dark. The absorbance was determined at 725 nm, using a Synergy HT Multi-detection microplate reader (Biotek, Bad Friedrichshall, Germany) running Biotek Gen 5™ software.

Gallic acid was used as the reference phenolic compound and the total phenolic content of the extracts was expressed in µg of Gallic acid equivalents (GAE)/mg dry extract, as Mean ± SD of two independent assays.

### 2.6. Determination of DPPH• Scavenging

The free radical 2,2-diphenyl-1-picrylhydrazyl (DPPH^•^) was used to determine the antioxidant potential of the extracts and the results were expressed as a percentage of scavenging [23]. Briefly, 25 µL of each extract, prepared in methanol at five final different concentrations (550.0, 870.0, 138.0, 69.0 and 34.7 µg/mL) were incubated with 200 µL of DPPH^•^ (Sigma-Aldrich, St. Louis, MO, USA) 100 µM solution (in methanol), for 5 min at room temperature and protected from light. The absorbance of the reaction product was determined at 515 nm using a Synergy HT Multi-detection microplate reader (Biotek, Bad Friedrichshall, Germany) running Biotek Gen 5™ software. A blank with methanol was included for each sample concentration.

### 2.7. Cell Assays

A murine macrophage cell line RAW 264.7 was grown at 37 °C in DMEM supplemented with glutamine, 10% inactivated fetal bovine serum (FBS), 100 U/L penicillin and 100 µg/mL streptomycin, under a 5% CO_2_ atmosphere. The cells were inoculated in 96-wells plates (Thermo Scientific, Carlsbad, CA, USA) and cultured until confluence. The dried extracts were dissolved in DMSO and kept at −20 °C until use. Four serial dilutions of the extracts were prepared in supplemented DMEM immediately before cell exposure. To determine the effect of the extracts in NO production by RAW 264.7 cells, a pre-treatment (2 h) with extract dilution or vehicle was undertaken, following the addition of 1 µg/mL lipopolysaccharide (LPS) from *Escherichia coli* (or vehicle) and incubated for 22 h at 37 °C, under a 5% CO_2_ atmosphere. The effect of the extracts in NO production was also evaluated in the absence of LPS, to observe possible changes in the NO basal levels. No LPS was added to the negative controls. Four independent assays were performed in duplicate [21].

### 2.8. Toxicity

*Bacopa* extracts’ toxicity was determined by assessing cell viability in the RAW 264.7 cells through the MTT assay described by Barbosa et al. [24]. After the incubation period (35,000 cells/well, 24 h at 37 °C), the RAW 264.7 cells were incubated for 45 min with MTT (0.5 mg/mL prepared in DMEM), which was converted by dehydrogenases of metabolically active cells from a yellow salt into an insoluble purple formazan product. The pellet was further solubilized with 100 µL DMSO and the absorbance measured at 510 nm in a microplate reader (Biotek, Bad Friedrichshall, Germany) operated using Biotek Gen 5™ software.

### 2.9. Anti-Inflammatory Potential

Macrophage cells release NO into the culture medium, which is converted into different nitrogen derivates, of which nitrite is stable, and thus, easy to measure as an indicator of NO production. The anti-inflammatory potential of the extracts from the wild populations of *B. monnieri* was predicted by evaluating their capacity to reduce NO produced by the RAW 264.7 macrophages, following inflammatory stimulation, after the incubation period, of the different extracts at different concentrations, in four serial dilutions from a stock of 200 μg dry extract/mL (24 h, 37 °C, 35,000 cells/well). NO was measured in the culture medium with 75 μL of Griess reagent (Sigma-Aldrich, St. Louis, MO, USA), mixed with 75 μL cell supernatant and incubated in the dark for 10 min. The absorbance was determined at 562 nm. Control values were obtained without the extracts and by adding LPS (1 μg/mL). The effect of the extracts on NO produced by the RAW 264.7 cells was also evaluated in the absence of LPS to obtain the levels of basal NO produced by the untreated cells. Dexamethasone was used as the positive control. At least four independent experiments were performed in duplicate. Results were expressed as the percentage of NO versus the untreated control [24].

### 2.10. Zebrafish Nile Red Fat Metabolism Assay

*B. monnieri* extracts were dissolved in DMSO at a 10 mg/mL concentration and stored at −20 °C until analysis. The extracts’ anti-obesity capacity was analyzed through the zebrafish Nile red assay, as previously described in [25]. Fluorescence intensity in the individual zebrafish larvae was quantified using ImageJ (http://rsb.info.nih.gov/ij/index.html accessed on 25 November 2019).

### 2.11. Statistical Analysis

IBM SPSS STATISTICS software v. 25.0 (IBM Corporation, New York, NY, USA, 2011) was used to determine the profiles of the carotenoids and chlorophylls. Data were analyzed for normality and homogeneity of variances using Kolmogorov–Smirnov’s and Leven’s tests. The amounts of compounds 1, 2, 5–10, 14, 15, 18, 19, the total pigments, total carotenoids and total chlorophylls within the analyzed samples were compared using one-way ANOVA with Tukey’s HSD (honestly-significant-difference) as a post hoc test. The amounts of compounds 11, 12, 13, 12 + 13, 16 and 17 were compared through an unpaired t-test. The quantity of pigments in each sample (in μg/g of lyophilized biomass) was presented as the mean ± SD of at least three independent experiments. For the analysis of the lipid-reducing activity data, a one-way ANOVA analysis of variance was used followed by a Dunnett post hoc test.

## 3. Results

### 3.1. Identification of Bacopa monnieri using Molecular Data

Our ITS, *trn*L-*trnF* and *rbc*L partial sequences were compared with those in the NCBI using BLAST alignment and the results are shown in Table 3.

The percentages of the sequence identity ranged from 99.1 to 100%, which indicates that within these genetic regions, the Mexican plant studied is molecularly validated as the taxon *B. monnieri.*

### 3.2. Carotenoids and Chlorophylls Profiling

**Figure 2:**
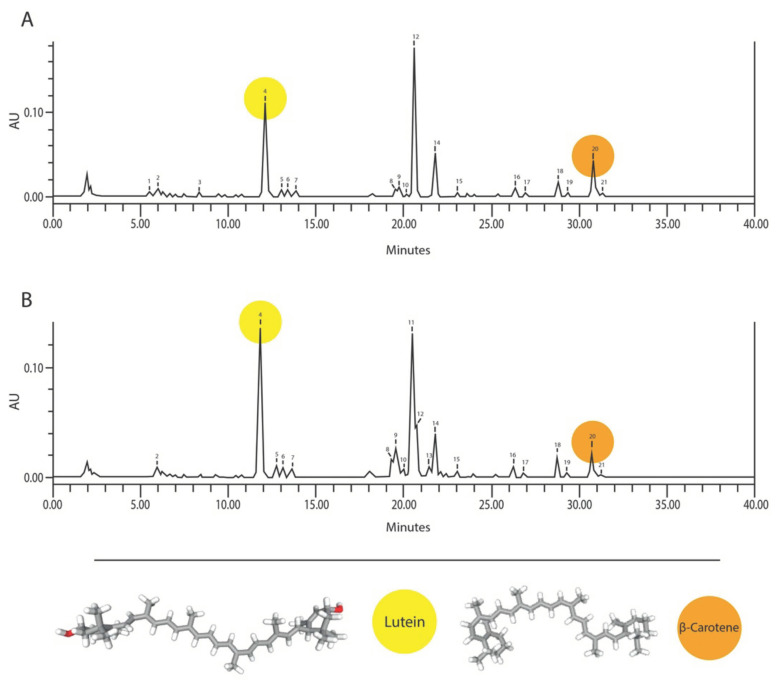
Carotenoid and chlorophyll profiles of methanol extracts of wild *B. monnieri* from different environments: BH (**A**) and BX (**B**). HPLC-PDA recorded at 450 nm. β-Carotene derivative (1, 5–7), Violaxanthin derivative (2–3), Chlorophyll b derivative (8–10, 14), Unidentified chlorophyll (12–13, 16–19), Lutein (peak number 4, yellow circle), Chlorophyll *b* (11), Chlorophyll *a* (15), β-Carotene (peak number 20, orange circle) and 13-cis-β-Carotene (21). The chemical structure model of Lutein and β-Carotene were taken from PubChem, NCBI.

An HPLC-PDA analysis of the extracts of the *B. monnieri* allowed the identification and quantification of 21 compounds comprising 12 chlorophylls and nine carotenoids. In Figure 2A,B, only the two most contrasting chromatograms are shown. Table 4 shows the pigments’ profile obtained from four different extracts. Sample BX had the highest pigment content, 53.512 ± 0.826 μg/mg dry extract (DE), followed by BE (42.654 ± 0.054 μg/mg DE), while BH and BS had the lowest amount (41.741 ± 1.953 and 37.675 ± 0.051 μg/mg DE, respectively), *p* < 0.05 (ANOVA and Tukey’s HSD).

The same trend was observed in all the samples for total carotenoids and chlorophylls, with the latter in higher amounts. Regarding the total chlorophylls, its concentration varied between 36.013 ± 0.055 (BS) and 51.919 ± 0.863 (BX) (Table 4). The chlorophyll *b* (peak 11, Table 4) had higher concentrations in BH and BE, with 25.053 ± 0.939 and 26.419 ± 0.026 μg/mg DE, respectively.

In addition, lutein (Figure 2A,B; peak 4, Table 4) was the most abundant carotenoid, with amounts ranging from 0.921 ± 0.031 (BH) to 1.149 (BX) μg mg^–1^ of DE, followed by β-carotene (Figure 2A,B; peak 20, Table 4) with amounts ranging from 0.095 ± 0.003 (BX) to 0.198 ± 0.006 (BS) μg mg^–1^ DE. A violaxanthin derivate and Ɛ-carotene derivatives were also tentatively identified in all the samples.

The compounds **12** and **13**, corresponding to unidentified chlorophylls, were not detected in the BH extract (Figure 2A). Meanwhile, in the BX extract, the undetected peaks were 1 and 3, corresponding to the Ɛ-carotene derivative and violaxanthin derivative, respectively. BX was also the only extract where compound **12** was detected as an unidentified chlorophyll (Figure 2B).

### 3.3. Total Phenolic Content

The total phenolic content (TPC) found in the four wild populations of *B. monnieri* ranged from 54.8 ± 5.8 (BX) to 70.3 ± 2.2 (BS) µg GAE/mg dry extract. BS had the highest concentration of phenolic compounds with a value of 70.3 ± 2.2 µg GAE/mg dry extract; BH and BE showed a similar TPC with a value of 67.6 μg GAE/mg dry extract (Table 5).

### 3.4. Antioxidant Potential

The antioxidant potential of the different plant extracts was determined by evaluating their DPPH free radical scavenging capacities using an in vitro cell-free assay. The BH sample reached IC_50_ for DPPH radical scavenging at a value of 130.6 ± 3.0 µg dry extract/mL, followed by BX with a value of 132.0 ± 17.8 µg dry extract/mL; BE and BS presented higher values of 218.2 ± 6.0 and 249.9 ± 12 µg dry extract/mL, respectively (Table 5). The different compounds present in the extracts, including carotenoids and phenols, which are known for their ability to scavenge free radicals, may have contributed to the observed IC_50_ values.

**Figure 3:**
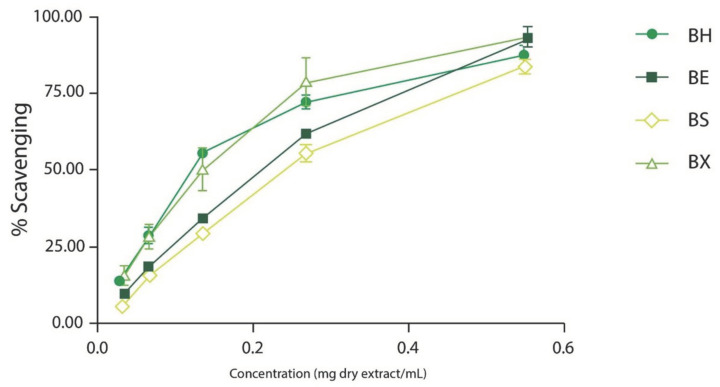
DPPH radical scavenging activity. Results are expressed as the mean ± SD of three independent experiments performed in duplicate, face to untreated control.

The BH extract only needed about 0.2 mg dry extract/mL to inhibit DPPH scavenging by about 90%, compared to BS, which inhibited DPPH scavenging by 80% with 0.5 mg dry extract/mL. BE and BX also inhibited about 90% of the DPPH radical scavenging with 0.5 mg of dry extract/mL (Figure 3).

### 3.5. Anti-Inflammatory Potential

**Figure 4:**
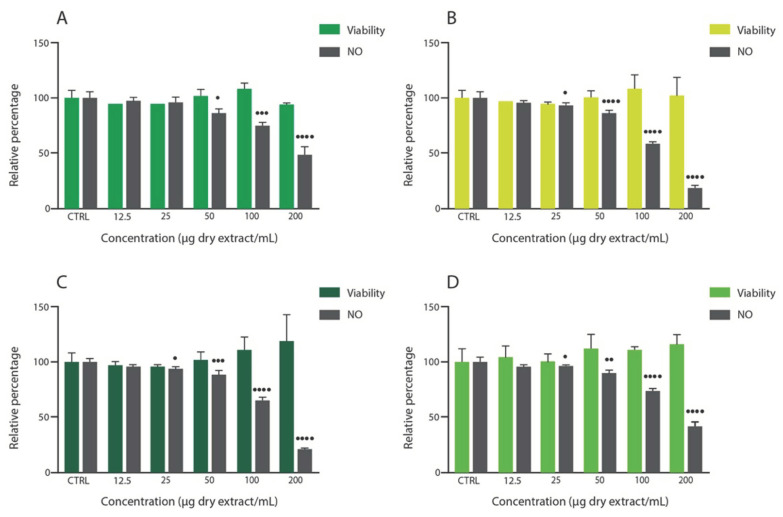
(**A**–**D**) Relative percentage of NO production by RAW 264.7 and the viability of cells in the presence of *B. monnieri* extracts at different concentrations. (**A**) BH, (**B**) BS, (**C**) BE and (**D**) BX. Results are expressed as a % of NO production and cell viability relative to the control. Results are expressed as the mean ± SD of at least four independent assays. • *p* < 0.05, •• *p* < 0.01, ••• *p* < 0.001, •••• *p* < 0.0001 (ANOVA, Tukey HSD).

The anti-inflammatory potential of *B. monnieri* was explored by analyzing its effect on the levels of NO produced by RAW 264.7 macrophages after stimulation with LPS. The BS and BE extracts (Figure 4B,C) showed a significant decrease in NO, starting from 25 to 200 μg dry extract/mL. For BX, the activity was from 50 to200 μg dry extract/mL, whereas the results for BH were very similar to those for BX (Figure 4A,D). For the highest concentration tested (200 μg/mL), the maximum inhibition of NO production was 48%, 18%, 22% and 41% for BH, BS, BE and BX, respectively.

### 3.6. Lipid-Reducing Activity in Different Extracts

**Figure 5:**
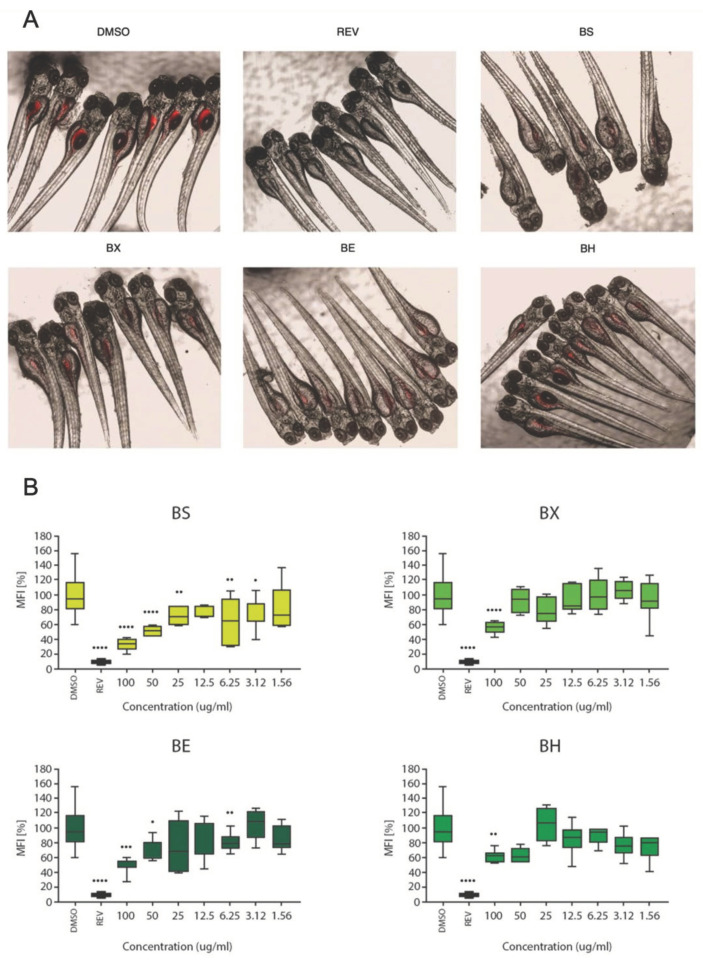
(**A**) Zebrafish Nile red fat metabolism assay representation with a strong fluorescence signal from the solvent control (DMSO) around the yolk sac and stomach in the zebrafish larvae. BS, BE, BX and BH fractions decreased Nile red staining at the highest concentration tested (100 µg/mL). (**B**) Quantification of the lipid-reducing activity in the zebrafish Nile red fat metabolism assay after 48 h exposure. A solvent control (0.1% dimethyl sulfoxide, DMSO) and positive control (50 µM resveratrol, REV) were included in the bioassay. Six to eight individual larvae per treatment group were used (n = 6–8). Values are expressed as mean fluorescence intensity (MFI) relative to the solvent control group. The data are represented as box-whisker plots from the 5th to 95th percentiles and statistically significant differences are shown with bullets (•••• *p* < 0.0001, ••• *p* < 0.001, •• *p* < 0.01, • *p* < 0.05).

The zebrafish Nile red fat metabolism assay was used to characterize the lipid-reducing activity of the four wild populations of *B. monnieri* extracts. The exposure of zebrafish larvae to all the extracts was positive and in the following descending order: BS, BX, BE and BH. Exposure resulted in significant neutral lipid-reducing activity in this assay after 48 h (Figure 5A). A considerable decrease in Nile red staining was observed for BS at 100, 50, 25, 6.25 and 3.13 µg/mL; for BX and BH only at 100 µg/mL; for BE at 100 and 50 µg/mL (Figure 5B).

Toxicity and malformations in the zebrafish larvae exposed to these four extracts were evaluated considering general toxicity (death after 24 or 48 h) and malformations in the morphological characteristics of the larvae. These adverse effects were not observed for all the extracts. Resveratrol (REV) was used as a positive control at a final concentration of 50 µM and significantly reduced the Nile red lipid staining in all the bioassays. The solvent control, 0.1% DMSO (dimethyl sulfoxide), did not cause any toxicity or observable malformations in the larvae.

## 4. Discussion

The Mexican plant studied in this work was validated as *Bacopa monnieri,* as it was found to be highly homologous with the barcoded DNA sequences of *B. monnieri* from Bangkok, Thailand [26]. It is worth mentioning that the BOLDSYSTEMS record of *B. monnieri* (GBVX639715) from Delhi, India [27], also matches the Mexican plant studied. The *exsiccatae* were submitted to the IZTA with voucher numbers 45166, 45167 and 45168 from the BH, BX and BS wild populations, respectively.

Based on the Britton and Khachik [28] scale, the β-Carotene concentration (mg/100 g) in plants can be low (0–0.1), moderate (0.1–0.5), high (0.5–2.0) and very high (>2.0). In general, green leafy vegetables have a moderate to very high concentration of β-Carotene. This information applied to all the results from this study of the Mexican plant; the mean, in mg, of the compound on a dry biomass corresponded to the category of very high for BE (38.7 mg/g), BH (33.8 mg/g), BS (32.2 mg/g) and BX (12.7 mg/g).

Dhami and Cazzonelli [29] recognized that carotenogenesis is affected by the changing environment; carotenoids and carotenoid derivatives play a role in plants’ environmental sensing and acclimatization. This information allows us to explain the reason why our results, although they have no difference in the total quantification of the total carotenoids, have a different proportion (Table 4). Samples collected in environments with high temperatures (20.5–50 °C) have a higher amount of β-Carotene; for the samples from Jalisco, BE had 0.168 μg/mg and BS had 0.198 μg/mg, the sample from Hidalgo, BH, had 0.175 μg/mg. Meanwhile, for the plants from Puebla, which grew under temperate conditions (21–28 °C), the BX sample had 0.095 μg/mg of β-Carotene, the lowest concentration, but had the highest amount of lutein (1.149 μg/mg), which constituted the total pigment content of 53.512 (μg/mg).

Moreover, in *Arabidopsis*, lutein and β-Carotene are the main carotenoids in mature leaves, accounting for almost 50% and 25% of the foliar carotenoids, respectively [30]. For *B. monnieri* from the Mexican plant, lutein and β-Carotene were also dominant; lutein values were close to 60% in BH, BE and BS, and 72% in BX. On the other hand, the β-Carotene values ranged from 5.7 to 11.9% in the Mexican plant, rather than the 25% mentioned above. No information on the carotenoid profile of *B. monnieri* has been found in the literature.

Mihaylova et al. [31] researched the presence of lutein in different plants for medicinal and edible use, from trace amounts to 208 µg/g DW, the highest amount found in *Silene vulgaris* (Bladder campion). Similar values were recorded in BE (226.6 µg/g DW), followed by BH, BS and BX (177.8, 157.7 and 152.7 µg/g DW), respectively. Recent studies indicate the health benefits of taking 10 mg/day of lutein [31]. On the other hand, in the food industry, color is an essential characteristic for the success of a product; the alternative to artificial colorants has been the use of carotenoids of natural origin. One of the plants with the most remarkable presence in the industry is *Tagetes erecta* (Marigold, Cempasúchil), native to Mexico, which manages to obtain 70 to 120 mg/g of xanthophylls, of which 80–90% corresponds to lutein [32]. Therefore, based on our results, we propose using the Mexican populations of *B. monnieri* as a natural resource, since the concentrations of lutein and carotenoids found in *B. monnieri* extracts are high and at levels comparable to other plant resources currently used in the food industry and traditional medicine.

Research on *B. monnieri* [33] in a swamp near Tuxpan, Ver. (Mexico), showed 7.69 ± 0.025 GAE mg/g extract, identical to the values obtained here for BX (7.29 GAE mg/g extract), followed by BS, BH and BE with 11.43, 13.05 and 15.54 GAE mg/g extract, respectively. On the other hand, wild populations of *B. monnieri* from different Indian locations report values from 5.67 ± 0.64 to 9.11 ± 0.19 GAE mg/g extract [34]. Our results using the Mexican plants had twofold or threefold concentration increases.

All the extracts of the *B. monnieri* Mexican plants were a good source of antioxidants. Similarly, following the example of the Indian species, which have an antioxidant potential close to 80% of DPPH radicals scavenging [35], the Mexican plants of *B. monnieri* can be used in the pharmacological and food industries due to their antioxidant properties, which have benefits for human health.

Inflammation is a multifactorial process in which diverse immune cells and mediators are involved, through which the host responds to stimuli with the aim of restoring the homeostasis of the organism and promoting tissue repair. In this survey, we have focused on exploring the effects of the *B. monnieri* extracts on the metabolism of macrophages, namely the inflammatory mediator NO, whose modulation can contribute to preventing the onset and progression of inflammation. The anti-inflammatory potential of the *B. monnieri* extracts was analyzed in RAW 264.7 after stimulation with LPS, by assaying their effect on the levels of NO produced by the macrophages. The IC_50_ found for the different extracts can divide the samples into two groups, with a significant difference between them (Table 5). The first group includes BE and BS, showing IC_50_ values of 122.4 ± 1.6 and 134 ± 3.8 µg dry extract/mL, respectively, while the second group, BX and BH, had IC_50_ values of 174.4 ± 7.0 and 181.7 ± 14.3 µg dry extract/mL, respectively (Table 5). This supports the inclusion of this plant resource in the Pharmacopoeia for its bioactive compounds, such as carotenoids, which are capable of managing diseases with an associated inflammatory process [3].

The decrease in NO levels observed here may correlate with the compounds present in the extracts, or with the synergistic interaction between the pigments and phenols. Lutein was previously described to decrease NO production in LPS-stimulated RAW 264.7 macrophages by reducing iNOS gene expression at the mRNA level [36], suggesting a similar potential anti-inflammatory contribution to that observed with the *B. monnieri* extracts (Table 4 and Table 5, Figure 3). Recent works have shown the beneficial effects of crude extracts of *B. monnieri* from India in preventing dementia by their anti-inflammatory action in male Wistar rats [37]. However, the higher lutein content did not mean a higher anti-inflammatory potential, which emphasizes the possible synergic effect of other pigments and compounds present in the extracts analyzed.

Cell viability was not affected under the concentrations tested, demonstrating the non-toxic behavior of the extracts. In a study conducted in male and female Sprague–Dawley rats, it was shown that acute and chronic administration of *B. monnieri* extract did not cause any signs of toxicity or other symptoms [38].

The zebrafish model has been used in numerous biomedical investigations to determine the biological and anti-obesity activity of different biomolecules [39]. For example, adipogenesis was decreased by *Zingiber officinale* [40]. Two chlorophyll derivatives showed significant neutral lipid-reducing activity in the zebrafish Nile red fat metabolism assay [41].

Our results showed a significant neutral lipid-reducing activity of the Mexican plant, particularly the BS extract, which also had the highest anti-inflammation activity. Obesity is also known as chronic low-grade inflammation [42]. Consumption of high concentrations of carotenoids can help reduce diseases caused by oxidative stress in the human body, although they might also inactivate free radicals [29]. Concordantly, it was shown that a high amount of β-Carotene displayed anti-obesity activity with effects on adipogenesis [43].

Wild Mexican *B. monnieri* extracts are rich in carotenoids, which may have contributed to the observed anti-obesity and anti-inflammatory activity. Our results agree with previous studies, in which the promising effects of extracts or fractions from different sources were observed, such as the effect of cyanobacteria on the intestinal lipid uptake of zebrafish [44,45] and the reduction of neutral lipids by seagrass [46] or by actinobacteria [47], but of which the responsible metabolites are still to be identified, as is the case with *B. monnieri*.

## 5. Conclusions

This study presented the pigment profile of *Bacopa monnieri* from Mexico and the evaluation of its lipid activity reduction for the first time. It verified that the different Mexican germplasms presented differential responses (chlorophyll and carotenoid). It was found that in different environmental conditions, the concentration of lutein and β carotene were modified.

The information gathered on the wild Mexican plant from the CMVB will allow for the selection of optimal germplasm-containing biocompounds with high antioxidant activity and carotenoid-rich, anti-inflammatory and anti-obesity potential. As shown in the bioassays performed, differential responses in the bioassays concerning the study area were also observed. These findings indicate new attributes of *B. monnieri*, which enriches the knowledge of this natural source of compounds. Therefore, they could be used as a raw material of excellent quality to improve nutritional and medicinal supplements, just as germplasms from other regions of the world have been used, placing itself in the international market for natural products.

## Figures and Tables

**Table 1:** Oligonucleotides used for PCR and sequencing of ITS, *trn*F-*trn*L (B49317-A50272) and *rbc*L, for wild Mexican *B. monnieri*.

Oligonucleotide/Reference	Sequence (5′ to 3′)
ITS1/[18]	TCCGTAGGTGAACCTGCGG
ITS4/[18]	TCCTCCGCTTATTGATATGC
B49317/[19]	SCGAAATCGGTAGACGCTACG
A50272/[19]	ATTTGAACTGGTGACACGAG
*rbc*L-F/[20]	ATGTCACCACAAACAGAAACTAAAGC
*rbc*L-R/[20]	AATTCAAATTTAATTTCTTTCC

**Table 2:** Calibration curves of authentic standards used for quantification of different Carotenoids and Chlorophyll-*a*.

Standards	Calibration Curve	r^2^	LOD (μg/mL) ^1^	LOQ (μg/mL) ^2^
Violaxanthin	y = 1,152,598,695x – 21,356	0.9997	0.3877	1.1748
Lutein	y = 141,092,914x + 5527	0.9998	0.2867	0.8688
ε-Carotene	y = 226,925,025x – 83,662	0.9996	2.3099	6.9998
Chlorophyll-a	y = 7,471,178x + 2673	0.9998	1.4721	4.4608
β-Carotene	y = 290,231,487x + 172,758	0.9997	1.9354	5.8649

^1^ LOD: limit of detection. ^2^ LOQ: limit of quantification.

**Table 3:** Taxonomic identification of *B. monnieri* from Mexico using molecular marker sequences compared to reference materials.

Molecular Marker	Length of Sequence (pb)	Query Cover (%)	Percentage of Identity (%)	GenBank Accession Number	IZTA Herbarium	Origin
ITS	642	99	99.1	**OL451230**/KM887387	45166	**Hidalgo**/Delhi
*trn*L-*trn*F	955	100	99.9	**OL456239**/LC310979		**Hidalgo**/Bangkok
*rbc*L	1266	100	100	**OL456230**/LC214987		**Hidalgo**/Bangkok
ITS	432	100	100	**OL451229**/KR215626	45167	**Puebla**/Delhi
*trn*L-*trn*F	955	100	99.9	**OL456238**/LC310979		**Puebla**/Bangkok
*rbc*L	1266	100	100	**OL456234**/LC214987		**Puebla**/Bangkok
ITS	703	99	99.6	**OL451228**/KM887387	45168	**Jalisco**/Delhi
*trn*L-*trn*F	955	100	99.7	**OL456237**/LC310979		**Jalisco**/Bangkok
*rbc*L	1266	100	100	**OL456233**/LC214987		**Jalisco**/Bangkok

In bold Mexican plant.

**Table 4:** Carotenoid and Chlorophyll content (μg/mg dry extract) of methanol extracts of *B. monnieri* obtained from different environments in Mexico determined using HPLC-PDA ^1^.

Peak	Compound	RT (min)	BH	BE	BS	BX
1	ε-Carotene derivative	5.6	0.072 ± 0.001 ^a^	0.061 ± 0.002 ^b^	0.052 ± ≤0.001 ^c^	nd
2	Violaxanthin derivative	6.0	0.012 ± ≤0.001	0.012 ± ≤0.001	0.012 ± ≤0.001	0.011 ± ≤0.001
3	Violaxanthin derivative	8.4	0.005 ± ≤0.001	0.004 ± ≤0.001	0.004 ± ≤0.001	nd
4	Lutein	12.2	0.921 ± 0.031 ^b^	0.988 ± 0.004 ^b^	0.969 ± 0.002 ^b^	1.149 ± 0.029 ^a^
5	ε-Carotene derivative	13.0	0.073 ± ≤0.001 ^b^	0.088 ± 0.002 ^a, b^	0.076 ± 0.006 ^a, b^	0.090 ± 0.003 ^a^
6	ε-Carotene derivative	13.4	0.070 ± 0.001	0.076 ± 0.001	0.073 ± 0.006	0.078 ± 0.002
7	ε-Carotene derivative	13.9	0.067 ± 0.001	0.068 ± 0.003	0.070 ± 0.004	0.072 ± ≤0.001
8	Chlorophyll b derivative	18.3	0.210 ± 0.019 ^b^	0.226 ± 0.001 ^b^	0.249 ± 0.007 ^b^	1.149 ± 0.068 ^a^
9	Chlorophyll b derivative	19.7	2.443 ± 0.2503 ^b^	1.724 ± 0.100 ^c^	1.342 ± 0.121 ^c^	6.759 ± 0.027 ^a^
10	Chlorophyll b derivative	20.0	0.197 ± 0.035 ^c^	0.376 ± 0.028 ^b^	0.343 ± 0.003 ^b^	0.862 ± 0.014 ^a^
11	Chlorophyll b	20.5	25.053 ± 0.939 ^a, b^	26.419 ± 0.026 ^a^	23.948 ± 0.223 ^a, b^	23.783 ± 0.798 ^b^
12	Unidentified chlorophyll	20.7	nd	Nd	nd	7.467 ± 0.064-
13	Unidentified chlorophyll	21.3	nd	0.396 ± 0.056 ^c^	0.642 ± 0.060 ^b^	1.280 ± 0.009 ^a^
14	Chlorophyll b derivative	21.9	7.301 ± 0.466 ^a^	6.063 ± 0.203 ^b, c^	4.552 ± 0.124 ^c, d^	5.023 ± 0.109 ^c^
15	Chlorophyll a	23.1	0.522 ± 0.011 ^c^	0.566 ± 0.011 ^b, c^	1.097 ± 0.007 ^a^	0.630 ± 0.042 ^b^
16	Unidentified chlorophyll	26.3	1.393 ± 0.114 ^b^	2.064 ± 0.084 ^a^	1.275 ± 0.074 ^b^	1.535 ± 0.063 ^b^
17	Unidentified chlorophyll	26.9	0.546 ± 0.036	0.527 ± 0.035	0.374 ± 0.012	0.507 ± 0.074
18	Unidentified chlorophyll	28.8	2.307 ± 0.036 ^b^	2.440 ± 0.043 ^b^	2.076 ± 0.058 ^c^	2.642 ± 0.009 ^a^
19	Unidentified chlorophyll	29.4	0.365 ± 0.078	0.380 ± 0.042	0.309 ± 0.080	0.374 ± 0.027
20	β-Carotene	30.8	0.175 ± 0.007 ^b^	0.168 ± 0.002 ^b^	0.198 ± 0.006 ^a^	0.095 ± 0.003 ^c^
21	13-cis-Beta- Carotene	31.4	0.004 ± 0.002	0.003 ± ≤0.001	0.007 ± 0.001	nq
Total carotenoids			1.575 ± 0.047	1.637 ± 0.005	1.662 ± 0.004	1.592 ± 0.038
Total chlorophylls			40.166 ± 1.906 ^b, c^	41.017 ± 0.060 ^b^	36.013 ± 0.055 ^c^	51.919 ± 0.863 ^a^
Total pigments			41.741 ± 1.953 ^b, c^	42.654 ± 0.054 ^b^	37.675 ± 0.051 ^c^	53.512 ± 0.826 ^a^

^1^ Values are expressed as the mean ± SD of two determinations (nq: not quantified, nd: not detected or under limit of detection, RT: retention time). Different superscript letters in the same row denote statistical differences at *p* < 0.05 (ANOVA, Tukey HSD).

**Table 5:** Total phenolic content (TPC) and IC_50_ values obtained for the antioxidant (DPPH• scavenging) and anti-inflammatory potential (NO reduction in RAW 264.7 cells) of the methanol extracts of *B. monnieri* obtained from different environments in Mexico *.

Sample	TPC (µg GAE/mg Dry Extract)	DPPH^•^ Scavenging (IC_50_, µg Dry Extract/mL)	NO Reduction in RAW 264.7 Cells (IC_50_, µg Dry Extract/mL)
BH	67.6 ± 4.3 ^a^	130.6 ± 3.0 ^a^	181.7 ± 14.3 ^b^
BE	67.6 ± 5.6 ^a^	218.2 ± 6.0 ^c^	134.7 ± 3.8 ^a^
BS	70.3 ± 2.2 ^a^	249.9 ± 12.1 ^d^	122.4 ± 1.6 ^a^
BX	54.8 ± 5.8 ^b^	132.0 ± 17.8 ^b^	174.4 ± 7.0 ^b^

GAE—Gallic acid equivalents. * Values are expressed as the mean ± SD of at least three independent experiments performed in duplicate. Different superscript letters in the same column denote statistical differences at *p* < 0.05 (ANOVA, Tukey HSD).

## Data Availability

All data generated or analyzed during this study are included in this published article.

## Supplementary Materials

The following supporting information can be downloaded at: https://www.mdpi.com/article/10.3390/biology12040620/s1, Figure S1: Carotenoid and chlorophyll profiles of methanol extracts of wild *B. monnieri* from different environments: BH (A), BX (B), BE (C), and BS (D). HPLC-PDA recorded at 450 nm. β -Carotene derivative (1, 5-7), Violaxanthin derivative (2-3), Chlorophyll b derivative (8-10, 14), Unidentified chlorophyll (12-13, 16-19), Lutein (peak number 4, yellow circle), Chlorophyll b (11), Chlorophyll a (15), β-Carotene (peak number 20, orange circle), 13-cis-β-Carotene (21).
