# Supplementary material for: Antioxidant, Anti-Inflammatory and Anti-Obesity Potential of Extracts Containing Phenols, Chlorophyll and Carotenoids from Mexican Wild Populations of Bacopa monnieri (L.) Wettst
Source: Biology (Basel). 2023 Apr 19;12(4):620. doi: 10.3390/biology12040620 (PMC10135869; doi:10.3390/biology12040620)
Supplement: Supplementary file 1 [file biology-12-00620-s001.zip › biology-2239947-supplementary/Figure S1.docx]

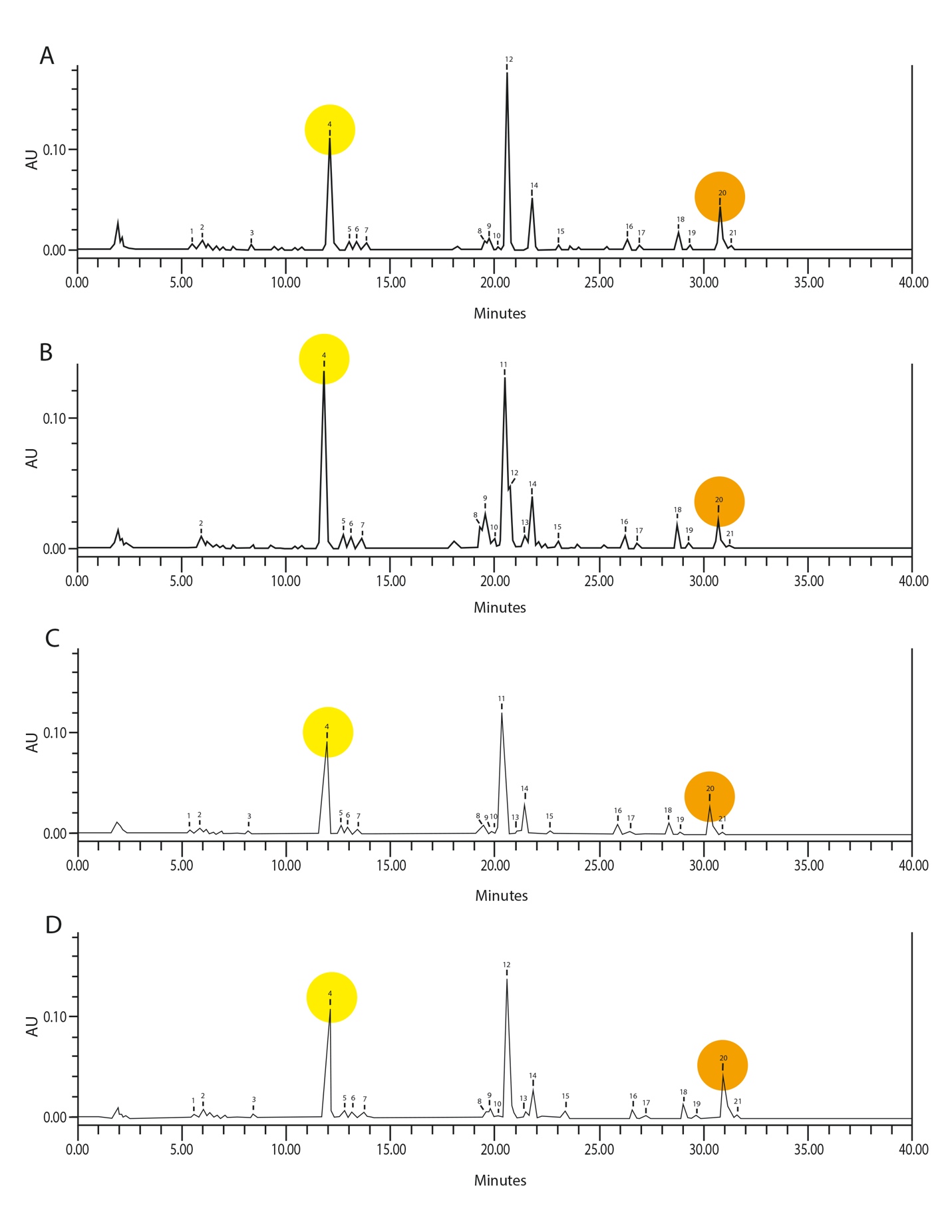


Figure S1. Carotenoid and chlorophyll profiles of methanol extracts of wild B. monnieri from dif-ferent environments: BH (A), BX (B), BE (C), and BS (D). HPLC-PDA recorded at 450 nm. β -Carotene derivative (1, 5-7), Violaxanthin derivative (2-3), Chlorophyll b derivative (8-10, 14), Unidentified chloro-phyll (12-13, 16-19), Lutein (peak number 4, yellow circle), Chlorophyll b (11), Chlorophyll a (15), β-Carotene (peak number 20, orange circle), 13-cis-β-Carotene (21).
